# Primary Gastro-Intestinal Lymphoma and Gastro-Intestinal Adenocarcinoma: An Initial Study of CT Texture Analysis as Quantitative Biomarkers for Differentiation

**DOI:** 10.3390/life11030264

**Published:** 2021-03-23

**Authors:** Lin Ding, Sisi Wu, Yaqi Shen, Xuemei Hu, Daoyu Hu, Ihab Kamel, Zhen Li

**Affiliations:** 1 Department of Radiology, Tongji Hospital, Tongji Medical College, Huazhong University of Science and Technology, Wuhan 430000, China; dinglin210@hust.edu.cn (L.D.); wss0331@126.com (S.W.); yq-shen@hust.edu.cn (Y.S.); mayjuly3720@163.com (X.H.); daoyuhu@hust.edu.cn (D.H.); 2 Russell H. Morgan Department of Radiology and Radiological Science, The Johns Hopkins Medical Institutions, Baltimore, MD 21105, USA; ikamel@jhmi.edu

**Keywords:** gastrointestinal tract, lymphoma, adenocarcinoma, diagnosis, multidetector computed tomography

## Abstract

Background: To explore the potential role of computed tomography (CT) texture analysis and an imaging biomarker in differentiating primary gastro-intestinal lymphoma (PGIL) from gastro-intestinal adenocarcinoma (GIAC). Methods: A total of 131 patients with surgical pathologically PGIL and GIAC were enrolled in this study. Histogram parameters of arterial and venous phases extracted from contrast enhanced modified discrete cosine transform (MDCT) images were compared between PGIL and GIAC by Mann–Whitney U tests. The optimal parameters for differentiating these two groups were obtained through receiver operating characteristic (ROC) curves and the area under the curve (AUC) was calculated. Results: Compared with GIAC, in arterial phase, PGIL had statistically higher 5th, 10th percentiles (*p* = 0.003 and 0.011) and statistically lower entropy (*p* = 0.001). In the venous phase, PGIL had statistically lower mean, median, 75th, 90th, 95th percentiles, and entropy (*p* = 0.036, 0.029, 0.007, 0.001 and 0.001, respectively). For differentiating PGIL from GIAC, V-median + A-5th percentile was an optimal parameter for combined diagnosis (AUC = 0.746, *p* < 0.0001), and the corresponding sensitivity and specificity were 81.7 and 64.8%, respectively. Conclusion: CT texture analysis could be useful for differential diagnosis of PGIL and GIAC.

## 1. Introduction

Primary gastrointestinal lymphoma (PGIL) is one of the most common sites of extranodal lymphoma, comprising about 30–40% of the patients [1,2]. Gastrointestinal adenocarcinoma (GIAC) is the most common type and constitutes nearly half of malignant lesions detected in the gastrointestinal tract [3,4]. The 5-year overall survival (OS) rate for primary gastric lymphoma is 79.5%, whereas for gastric adenocarcinoma is under 30% in most countries [5,6]. For primary intestinal non-Hodgkin’s lymphoma, the 5-year OS is 58%, but for small-bowel adenocarcinoma is 34.9% [7,8]. Differentiating between PGIL and GIAC is of great significance for determining choice of treatment and the subsequent prognosis and the clinical outcome.

Although some diagnostic examinations, including positron emission tomography (PET) [9], endoscopy and magnetic resonance imaging (MRI) [10], can be employed as facilitate diagnosis these two tumors, contrast-enhanced modified discrete cosine transform (MDCT) is still the dominant imaging modality to detect gastrointestinal (GI) neoplasms.

For PGIL, radiotherapy, chemotherapy and immunotherapy currently demonstrate superior response compared to surgical treatment, which is more appropriate for the patients accompanied by complications of lymphoma, perforation and massive bleeding for instance [11,12]. Moreover, these therapies carried a better 5-year survival rate in PGIL than in GIAC [13]. Surgical management of GIAC remains the first choice to avoid tumor progression or metastasis. Differentiating between PGIL and GIAC may avoid invasive surgery, and to prevent further the risk of perforation during chemotherapy and can help monitoring the progress of PGIL treatments. Although the identification of these tumors is characteristically made by pathology after operation or endoscopic-guided biopsy, the diagnosis and staging are typically directed by contrast enhanced computed tomography (CT). The typical appearance of GI adenocarcinoma is concentric lumen narrowing with irregular boundary, complete bowel obstruction, and heterogeneous enhancement; on the other hand, the representative appearance of GI lymphoma is coarse segmental wall thickening with ulceration and necrosis, aneurismal dilatation of gastrointestinal wall [14]. PGIL may have various appearances, which can be explained by the nature of lymphocytes and different ways they develop and spread. Thus, the radiographic appearance of PGIL is variable. It is a challenge for contrast enhanced CT to precisely distinguish these two kinds of neoplasms.

One possible resolution for solving this problem is CT-based texture analysis of the contrast enhanced CT data set. Texture analysis can extract texture information in a quantitative manner, in the way of converting changes in pixel intensity into mathematical form. It can enhance the value of contrast enhanced CT examinations and facilitate in differentiating GI tumors non-invasively. CT texture analysis detects slight differences in pixels, as well as evaluates tumor heterogeneity, which provides the information of the tumor microenvironment indirectly [15]. One recent study demonstrated that CT texture analysis plays an important value in evaluating intestinal tumors [16]. 

We analyzed the images of both the arterial and venous phase to evaluate this effect. The purpose of our study was to determine whether CT-based texture analysis may be useful in differentiating PGIL from GIAC.

## 2. Materials and Methods

### 2.1. Patients Enrolled

**Figure 1:**
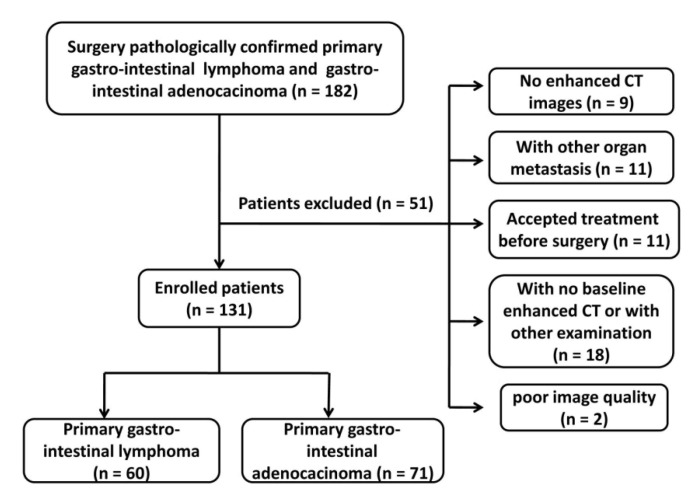
Flowchart of the study population. A total of 182 patients with surgically and pathologically diagnosed primary gastro-intestinal lymphoma (PGIL) or gastro-intestinal adenocarcinoma (GIAC) were enrolled, and finally 60 PGILs and 71 GIACs patients were enrolled in our study.

This retrospective study was approved by institutional review board of our hospital, and the requirement for informed consent was waived. A total of 182 patients with surgery pathologically confirmed PGIL or GIAC were enrolled from March 2013 to December 2019 in Tongji Hospital, Wuhan, China. Inclusion criteria of PGIL were based on Dawson standards [17]. Patients with PGIL or GIAC were included in our final cohort if they satisfy the following criteria: (1) newly diagnosed as having PGIL or GIAC; (2) aged >18 years; (3) had a definitive histological diagnosis through surgery or endoscopy; (4) no treatment was performed before CT examination; and (5) had an exclusion of other coexistent GI diseases; (6) adequate image acquisition and good image quality. Then, a total of 60 PGILs (38 male, 22 female; average age 50.75; range 24–74 years) and 71 GIACs (41 male, 30 female; average age 56.1; range 21–80 years) patients were enrolled in our study (Figure 1).

### 2.2. Image Acquisition

Patients were required to fast for 6 hours before the procedure and were persuaded into drinking 1500–2000 mL water prior to CT scan. All patients underwent dual phase contrast-enhanced CT using a 64-slice MDCT scanner (Discovery CT750 HD, GE Healthcare, USA) in a supine, feet-first position on the CT table. Intravenous contrast media 370 mg I/mL iopromide (Ultravist 370, Bayer Schering Pharma, Berlin, Germany) was administered at a flow rate of 3.5 mL/s, followed by a 20 mL saline flush. The total contrast volume was 1.5 mL/kg. Contrast material was injected through the ante-cubital vein using a dual-head injector (Stellant, Medrad, CO, USA), each with an injection time of 20 s. The time of arterial phase scanning started at 20s after a threshold enhancement of 120 HU was achieved in the abdominal aorta by using a bolus tracking technique (Smartprep, GE Healthcare Technologies). Venous phase scanning was initiated 60s after the completion of arterial phase scanning.

The CT imaging parameters were as follows: automatic tube current; tube voltage,120 kV; rotation time, 0.5 s; detector pitch, 0.984:1; matrix, 512 × 512; table speed, 39.37 mm/rotation; and slice thickness/interval, 1.25 mm. The scan data were reconstructed using field of view (FOV) (50 cm) and STANDARD (STD) reconstruction kernel and 100% ASIR reconstruction algorithms. All images were transferred to the workstation (AW4.7, GE Healthcare) for subsequent quantitative and qualitative analysis.

### 2.3. Image Analysis 

All data sets were measured and analyzed by two abdominal radiologists with more than 5-years-experience who were blinded to the pathological results. All evaluations were performed independently, and any remaining ambiguity was resolved by consensus. Texture analysis was achieved on arterial and venous phases of CT images using advanced software (Fire Voxel, New York University, New York, NY, USA). The region of interest (ROI) was manually delineated along the edge of lesion based on maximal-slice on axial images (slice thickness 1.25 mm), excluding areas of peri-tumor blood vessels, peri-normal intestinal wall, intestinal contents, and adjacent organs. Texture parameters derived from CT images were as follows: A-mean, A-median, A-5th, A-10th, A-25th, A-50th, A-75th, A-90th and A-95th percentiles, A-kurtosis, A-skewness; A-entropy in arterial phase; V-mean, V-median, V-10th, V-25th, V-50th, V-75th, V-90th and V-95th percentiles, V-kurtosis, V-skewness, V-entropy in the venous phase, respectively.

### 2.4. Statistical Analysis

Statistical analysis was carried out using IBM SPSS 23.0 software (Chicago, IL, USA) and MedCalc (Medcalc Software, Mariakerke, Belgium). The measurement consistency between two radiologists was tested by using interclass correlation coefficient (ICC). Continuous variables were expressed as mean±standard deviation, and categorical variables were expressed as frequency (percentage). Normality was estimated using the Shapiro-Wilk test (P ≥ 0.05 indicates normal distribution). In order to differentiate between PGIL and GIAC, continuous variables were compared by the Student t test or Mann-Whitney U test. Bonferroni test were used for post-hoc pairwise comparisons. Receiver operating characteristic (ROC) curves were used to determine the optimal threshold, sensitivity, and specificity of significant parameters for identifying PGIL and GIAC. Binary logistic regression analyses were used to assess the potential determinants for combined diagnosis and obtain model prediction equation. The comparison between the area under the curve (AUC) was assessed by Z test; *p* < 0.05 was considered as statistically significant.

## 3. Results

### 3.1. Clinical Features of the Patients with PGIL and Those with GIAC

A total of 131 patients with surgically and pathologically diagnosed PGIL or GIAC were enrolled in this study. The primary mass locations of 60 PGIL were as follows: 35 stomach, 3 duodenum, 10 small intestine, 5 colon and 7 cecus. The primary mass locations of 71 GIAC were as follows: 16 stomach, 24 duodenum, 14 small intestine, 12 colon, 4 cecus and 1 appendix. The maximum diameter of the lesions in each patient varied from 1.0 to 6.55 cm with a mean of 2.49 cm in PGIL and from 1.0 to 4.0 cm with a mean of 1.96 cm in GIAC. The clinical and pathological data were summarized in Table 1. The locations and the pathological classification of PGIL were shown in Table 2.

### 3.2. Histogram of the Comparison of Parameters Between the Patients with PGIL and Those with GIAC

**Figure 2:**
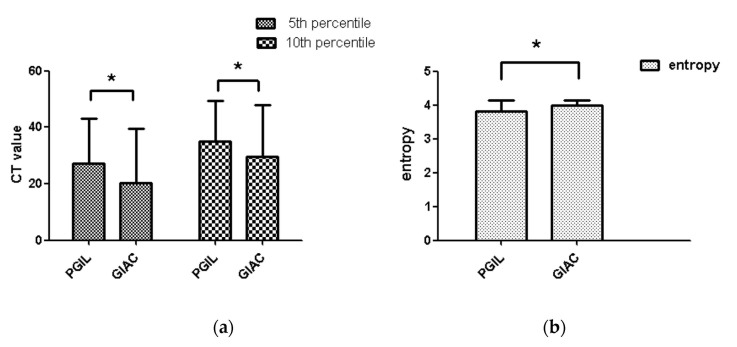
Histogram of the comparison of CTTA parameters between PGIL and GIAC in arterial phase; PGIL, primary gastrointestinal lymphoma; GIAC, gastrointestinal adenocarcinoma; * represents *p* < 0.05. (**a**) CT Value, (**b**) Entropy.

**Figure 3:**
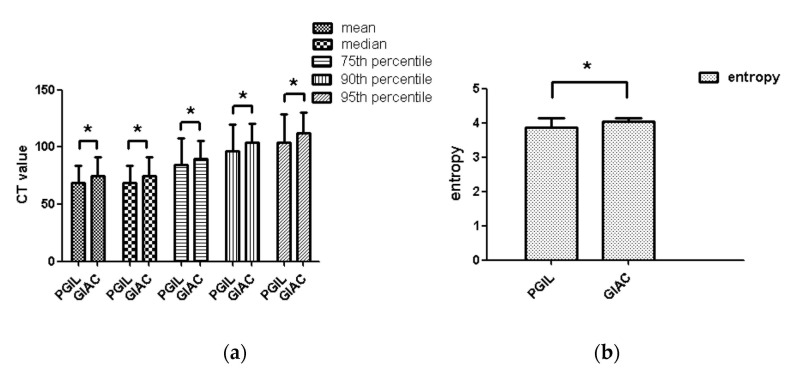
Histogram of the comparison of CTTA parameters between PGIL and GIAC in the venous phase; PGIL, primary gastrointestinal lymphoma; GIAC, gastrointestinal adenocarcinoma; * represents *p* < 0.05. (**a**) CT Value, (**b**) Entropy.

The degree of interobserver agreement was good (ICC >0.705) for all parameters in both arterial and venous phase (Supplementary Tables S1 and S2). As a result, this study chose a measurement value randomly from one radiologist analyzed as the final result. Compared with GIAC, PGIL had statistically higher 5th and 10th percentiles (*p* = 0.003 and 0.011) in arterial phase and statistically lower entropy (*p* = 0.001) (Table 3 and Figure 2). In the venous phase, PGIL had statistically lower mean, median, 75th, 90th, 95th percentiles and entropy (*p* = 0.036, 0.029, 0.007, 0.001 and 0.001, respectively) (Table 4 and Figure 3). However, there were no differences in arterial phase for mean, median, 25th, 50th, 75th, 90th, 95th percentiles, skewness and kurtosis between these two neoplasms (*p* = 0.681, 0.631, 0.126, 0.501, 0.488, 0.122, 0.055, 0.518 and 0.408, respectively), and in the venous phase for 5th, 10th, 25th, 50th percentiles, skewness and kurtosis between these two neoplasms (*p* = 0.550, 0.824, 0521, 0.087, 0.785 and 0.280, respectively)

**Figure 4:**
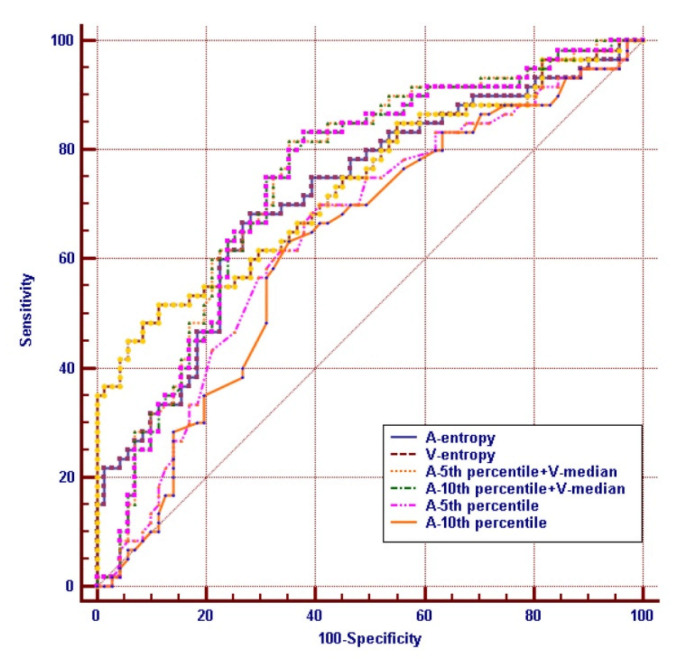
Receiver operating characteristic (ROC) analysis of every significant CTTA parameters. “+” present combined diagnosis. The A-5th+V-median shows the highest diagnosis efficiency, and its AUC is 0.746.

**Figure 5:**
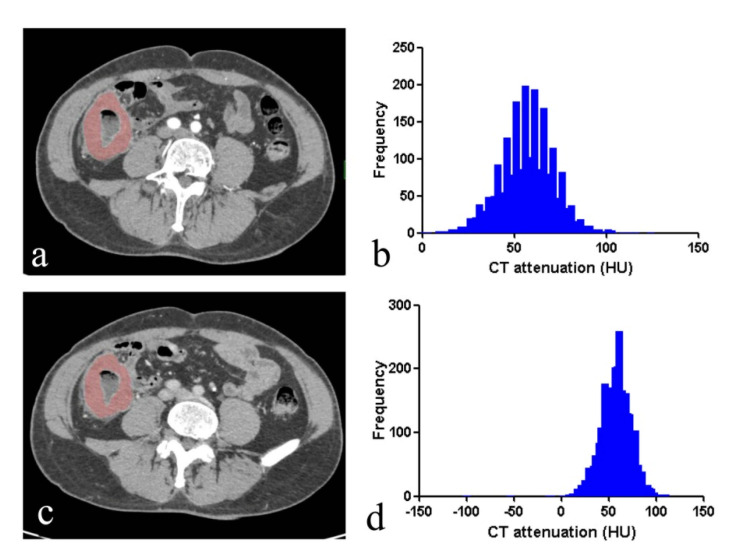
A 65-year-old male with primary intestinal non-Hodgkin lymphoma. CT images in axial arterial phase (**a**) and axial venous phase (**c**) show a coarse segmental wall thickening, with dilation of ileum with mild enhancement. The region of interest (ROI) was manually delineated along the edge of the lesion in arterial phase (**a**) and venous phase (**c**). Histograms of lesion in arterial phase (A-mean, 52.93 HU; A-5% percentile, 38HU; A-skewness, −0.32; A-kurtosis, 0.19; A-entropy, 3.99) (**b**) and venous phase (V-mean, 78.64 HU; V-median, 75HU; V-skewness, −0.13; V-kurtosis, 0.53; V-entropy, 3.89) (**d**) were shown.

**Figure 6:**
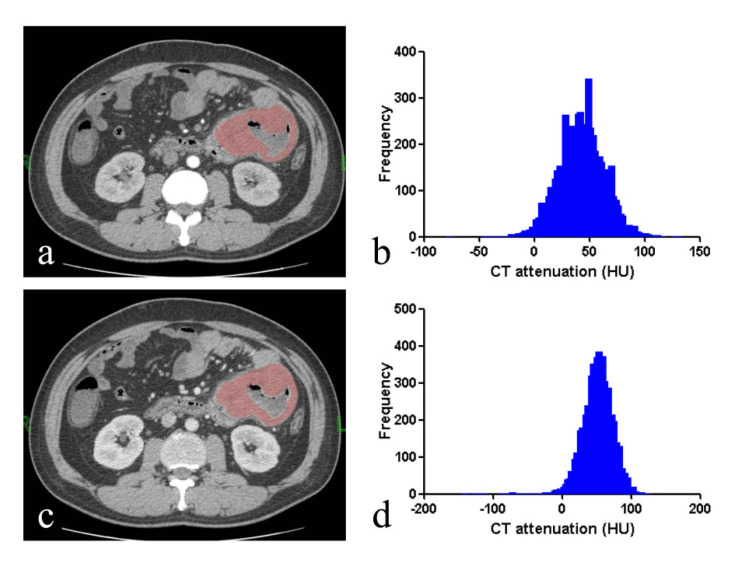
A 39-year-old male with intestinal adenocarcinoma. CT images in axial arterial phase (**a**) and axial venous phase (**c**) show concentric lumen narrowing with irregular edges with heterogenous enhancement. ROI was manually delineated along the edge of the lesion in arterial phase (**a**) and venous phase (**c**). Histograms of lesion in arterial phase (A-mean, 33.11 HU; A-5% percentile, 0HU; A-skewness, 0.002; A- kurtosis, 0.124; A-entropy, 3.959) (**b**) and venous phase (V-mean, 51.83 HU; V-median 55HU; V-skewness, 0.058; V-kurtosis, 0.001; V-entropy, 3.98) (**d**) were shown.

As shown in Table 5, A-5th, A-10th percentiles, A-entropy, V-mean, V-median, V-75th, V-90th, V-95th percentiles and V-entropy generated the higher AUC (AUC = 0.649; 0.628; 0.717; 0.607; 0.611; 0.636; 0.663; 0.671; 0.739, respectively) in differentiating PGIL from GIAC. Moreover, the combination of A-5th+V-median achieved the highest AUC values (0.746, *p* < 0.0001) (Figure 4), and the corresponding sensitivity and specificity were 81.7% and 64.8%, respectively. Representative cases of PGIL and GIAC were presented in Figure 5 and Figure 6.

### 3.3. Prediction of Differentiation PGIL from GIAC

Binary logistic regression analyses were performed. The likelihood ratio from the logistic regression model containing all the predictors were statistically significant (*p* < 0.05, Table 6), indicating that the model was able to distinguish between patients with PGIL and GIAC. 

The model prediction equation was as follows:

(1) $$P\;=\;\frac{1}{{1\;+\;e}^{-(-3.124\;+\;\left(-0.057\right)\;\times \;A5\%\;\mathrm{percentile}\;+\;0.065\;\times \;\mathrm{Vmedian})}}$$

## 4. Discussion

Some studies demonstrated that CT texture analysis is useful in evaluating clinical stage, prognosis and the response to therapeutic treatment in many categories of gastrointestinal tumors, such as esophageal cancers [18], gastrointestinal stromal tumors [19,20], and colorectal cancers [21,22]. CT texture analysis contributed complementary prognostic information to interim fluorodeoxyglucose positron emission tomography (FDG-PET) in lymphoma patients [23]. One recent study demonstrated that quantitative parameters obtained in dual-energy spectral computed tomography provided high accuracy for differentiating primary small intestinal lymphoma from small bowel adenocarcinoma [24]. Differentiating PGIL from GIAC before treatment is challenging, and this investigation has demonstrated that it is possible to achieve it by CT texture analysis. 

CT provides images with high spatial resolution in a very short time, together with its relative insensitivity to motion and breathing artefacts. Our results also illustrate it has highly consistent interclass correlation coefficient (ICC). The respiratory motion and intestinal peristalsis do not affect seriously the imaging quality and quantitative analysis. On contrast enhanced CT images, texture features reflect the distribution of the contrast agent between the intra- and extra-vascular extracellular space indirectly, which related to the effects of tumor vascular permeability. CT texture analysis is a reliable quantitative biomarker for the gastro-intestinal study [25,26,27].

In our study, there is no statistical difference in arterial phase for the mean or median CT value, and in the venous phase, the difference value for both mean and median CT attenuation between PGIL and GIAC is less than 6HU, which is statistically significant, reflecting the advantage of percentile and texture analysis. CT texture analysis may be a new quantitative biomarker to detect and provide more information for diagnosis.

Percentile in texture analysis has a good identifiable value, which is similar to the previous studies [16,19]. The reason for percentile in texture analysis been able to discriminate PGIL from GIAC is that CT attenuation corresponds to the degree of tumor enhancement, and higher attenuation demonstrates the degree of tumor enhancement, which may indicates the higher vascularity characterized by more aggressive tumors [21,28]. With regard to contrast-enhanced CT, the higher percentiles often reveal the blood perfusion in the lesion and the lower percentiles often indicate cystic degeneration and tissue necrosis. In the process of enhancement, both GIAC and PGIL showed a delayed enhancement pattern, but adenocarcinoma was more intensive than lymphoma, and with more necrotic composition.

In our study, higher percentiles (5th and 10th) in arterial phase and lower percentiles (75th, 90th and 95th) in the venous phase demonstrated statistically significant differences between PGIL and GIAC. This is an interesting discovery in our research. GIAC mainly involve the mucosa, whereas the PGIL mainly involve the submucosa, with distinct arterial blood supply, thus the time and range of enhancement are different. It was reported that small bowel adenocarcinoma usually shows moderate enhancement [29], whereas small intestinal lymphoma tends to be more homogenous and shows less enhancement [30]. Hypoxia and decreased angiogenesis are characteristically related to necrosis which could illustrate the greater heterogeneity and variability captured on contrast-enhanced CT imaging [31]. Tumor heterogeneity was positively correlated with hypoxia in biology. Texture analysis can not only quantify the spatial variations in parametric maps, but also provide supplementary information compared with histogram-based measures of parametric maps [32].

During our study, lower entropy in both arterial and venous phase demonstrated statistically differences between PGIL and GIAC. Some research has demonstrated that entropy can predict the histopathological characteristics of gastric cancers, and has shown to be a promising preoperative prognostic biomarker [28,33]. Entropy, the indicator of inhomogeneity, represents the irregularity of the voxel distribution, which suggests tumor heterogeneity caused by necrosis, angiogenesis, and cellular density [34]. A possible reason may be that lymphoma has less heterogeneity than adenocarcinoma, and the enhancement characteristics in both arterial and venous phase reflect the blood supply. Entropy represents overall tumor complexity and may be the best marker for the heterogeneity of the tumor [35] and has shown to be a promising quantitative biomarker to detect and provide more information for diagnosis.

In our present study, there was no statistically significant difference in skewness and kurtosis between PGIL and GIAC in both arterial and venous phase, reflecting a limited role of them in differentiating PGIL from GIAC. Texture parameters may show different significance depending on the method of analysis and the type of tumor. In a histogram, skewness evaluates the asymmetry of the probability distribution of a real-valued random variable [36]. The possible reason may be the enhancement for PGIL and GIAC is not very obvious, whereas the distribution of them is relatively uniform, and the histomorphology of the GI tract itself has its own characteristics. The skewness and kurtosis are higher order statics, although entropy is the first or second order statics [37]. This may illustrate why skewness and kurtosis perform differently from entropy. 

Our study indicated most CT texture analysis parameters had poor AUC values (<0.7), and the combination of A-5th+V-median generated the highest AUC (0.746) and limited accuracy (sensitivity 81.7%, specificity 64.8%) for distinguishing PGIL from GIAC. The existence of tissue necrosis and heterogeneity within these neoplasms, possibly render most CT texture analysis parameters ineffective while these characteristics may also the hallmarks for both PGIL and GIAC. We are interested to find out the sensitivity of the V-mean and the V-median is 80.0%, and the specificity of V-entropy is 88.7%, although the corresponding specificity and sensitivity are not satisfactory and the AUC value is not high overall, but in the actual diagnosis process, we can combine the CT texture analysis parameters with the clinical features for comprehensive diagnosis, rather than solely relying on CT texture analysis literature.

We established a binary regression model prediction equation based on A-5% percentile and V-median. As for the probability, if *p* < 0.5, PGIL would be more likely diagnosed, and if *p* > 0.5, GIAC would be considered first. Due to our limited number of patients, multicenter research with a large sample size may improve the regression prediction model equation diagnostic performance.

Two recent reviews summarized several quantitative imaging biomarkers which are useful in evaluating the treatment response and prognosis of gastric cancer, including apparent diffusion coefficient (ADC) from diffusion-weighted magnetic resonance imaging (DW-MRI), quantitative parameters from dynamic contrast-enhanced magnetic resonance imaging (DCE-MRI), as well as standardized uptake value (SUV) from 18F-fluorodeoxyglucose positron emission tomography/computed tomography (18F-FDG PET/CT) [38,39]. One recent study demonstrated that in patients with diffuse large B-cell lymphoma (DLBCL) of colon, volumetric FDG parameters of non-colon lesions make greater contribution to patient prognosis than FDG uptake of the colon lesion [40].

At present, the gastro-intestinal quantitative studies are difficult and rare. This research could provide a reference value for further studies with large samples and contribute to the development of CT texture analysis, but our study has several restrictions. First of all, the study population was relatively small and scattered, and we need to refine them in our next step. Second, this was a retrospective study, therefore subjective biases existed in patient selection. Third, this preliminary study only utilized the first-order parameters of CT texture analysis, radiomic or machine learning would be involved in the next prospective study of multi-center with a large sample size. By the way, due to some unique physiological characteristics (rapid transit, alkaline content and IgA secretion), small bowel neoplasms are rare and comprise merely 1–3% of all gastrointestinal malignancies [41], and the identification of diagnostic discriminating differences between them may be difficult.

## 5. Conclusions

CT texture analysis parameters, especially the V-median+A-5th percentile, can be used as a quantitative tool to differentiate PGIL from GIAC.

## Figures and Tables

**Table 1:** Characteristics of patients with PGIL and GIAC.

	PGIL	GIAC
**Number of patients**	60	71
**Age, y**	50.75 (24–74)	56.1 (21–80)
**Sex**		
**Man**	38 (63.3%)	41 (57.7%)
**Woman**	22 (36.7%)	30 (42.3%)
**Primary mass location**		
**Stomach**	35 (58.3%)	16 (22.5%)
**Duodenum**	3 (5%)	24 (33.8%)
**Small intestine**	10 (16.7%)	14 (19.7%)
**Colon**	5 (8.4%)	12 (17.0%)
**Cecus**	7 (11.6%)	4 (5.6%)
**Appendix**	0 (0%)	1 (1.4%)
**Maxium diameter (cm)**		
	2.49 (1.0–6.55)	1.96 (1.0–4.0)

PGIL, primary gastrointestinal lymphoma; GIAC, gastrointestinal adenocarcinoma.

**Table 2:** The locations and pathological classification of PGIL.

Lymphoma Classification	Stomach	Small Intestine	Colon	Total Number
**Diffuse large B-cell lymphoma**	25 (41.67%)	5 (8.33%)	10 (16.67%)	40 (66.67%)
**Mucosa associated lymphoid tissue lymphoma**	9 (15%)	3 (5%)	0 (%)	12 (20%)
**Burkitt lymphoma**	0 (0%)	1 (1.67%)	0 (%)	1 (1.67%)
**Extranodal NK/T cell lymphoma**	0 (%)	1 (1.67%)	0 (%)	1 (1.67%)
**Follicular lymphoma**	1 (1.665%)	1 (1.665%)	0 (%)	2 (3.33%)
**Mantle cell lymphoma**	0 (%)	1 (1.665%)	1 (1.665%)	2 (3.33%)
**Peripheral T cell lymphoma**	0 (%)	1 (1.665%)	1 (1.665%)	2 (3.33%)

**Table 3:** Computed tomography texture analysis (CTTA) parameters in differentiating PGIL from GIAC in arterial phase.

Parameter	PGIL	GIAC	*p* Value
A-mean ^a^	60.00 ± 12.40	60.90 ± 17.48	0.681
A-median ^a^	60.23 ± 12.65	61.10 ± 17.65	0.631
A-5%th percentile ^a^	27.22 ± 15.78	20.31 ± 19.13	0.003 *
A-10%th percentile ^a^	34.88 ± 14.64	29.59 ± 18.27	0.011 *
A-25%th percentile ^a^	46.94 ± 14.07	44.14 ± 17.63	0.126
A-50%th percentile ^a^	59.83 ± 14.57	59.84 ± 17.61	0.501
A-75%th percentile ^a^	72.93 ± 16.66	75.62 ± 18.40	0.488
A-90%th percentile ^a^	84.44 ± 18.70	89.27 ± 19.78	0.122
A-95%th percentile ^a^	91.88 ± 20.83	97.48 ± 20.60	0.055
A-skewness	−0.08 ± 0.18	−0.12 ± 0.24	0.518
A-kurtosis	0.21 ± 0.27	0.29 ± 0.63	0.408
A-entropy	3.82 ± 0.31	4.00 ± 0.13	0.001 *

^a^ Units of HU for CT value; * represents *p* < 0.05.

**Table 4:** CTTA parameters in differentiating PGIL from GIAC in the venous phase.

Parameter	PGIL	GIAC	*p* Value
V-mean ^a^	68.87 ± 14.84	74.60 ± 16.55	0.036 *
V-median ^a^	69.10 ± 14.85	75.06 ± 16.41	0.029 *
V-5%th percentile ^a^	37.87 ± 27.34	33.04 ± 21.10	0.550
V-10%th percentile ^a^	45.47 ± 25.97	42.13 ± 20.12	0.824
V-25%th percentile ^a^	58.13 ± 23.89	57.33 ± 18.07	0.521
V-50%th percentile ^a^	71.57 ± 22.78	73.73 ± 16.41	0.087
V-75%th percentile ^a^	84.73 ± 22.92	89.49 ± 16.21	0.007 *
V-90%th percentile ^a^	96.37 ± 23.26	103.87 ± 16.69	0.001 *
V-95%th percentile ^a^	103.9 ± 24.52	112.62 ± 17.96	0.001 *
V-skewness	−0.094 ± 0.211	−0.105 ± 0.233	0.785
V-kurtosis	0.226 ± 0.463	0.127 ± 0.364	0.280
V-entropy	3.871 ± 0.275	4.035 ± 0.095	0.001 *

^a^ Units of HU for CT value; * represents *p* < 0.05.

**Table 5:** Effectiveness of CTTA in differentiating PGIL from GIAC.

Parameters	AUC (95% Confidence Interval)	Cut-off	Sensitivity (%)	Specificity (%)	*p*-Value
**A-5th percentile ^a^**	0.649 (0.560–0.730)	22	70	59.15	0.0023
**A-10th percentile ^a^**	0.628 (0.540–0.711)	33	63.3	64.8	0.0093
**A-entropy**	0.717 (0.631–0.792)	3.96	68.33	71.83	<0.0001
**V-mean ^a^**	0.607 (0.517–0.691)	76.05	80.0	47.9	0.0321
**V-median ^a^**	0.611 (0.522–0.695)	76.0	80.0	47.9	0.025
**V-75th percentile ^a^**	0.636 (0.547–0.718)	87.0	73.33	53.52	0.0059
**V-90th percentile ^a^**	0.663 (0.575–0.743)	92.8	58.33	70.42	0.0008
**V-95th percentile ^a^**	0.671 (0.584–0.751)	100	58.33	71.83	0.0004
**V-entropy**	0.739 (0.656–0.812)	3.93	48.33	78.73	<0.0001
**V-median+ A-5th** **percentile**	**0.746 (0.663–0.818)**	**NS**	**81.7**	**64.8**	**<0.0001**
**V-median+ A-10th percentile**	0.739 (0.650–0.832)	NS	83.3	62.0	<0.0001

Data in parentheses are ranges. “+” present combined diagnosis. Optimal area under the curve (AUC) values are in bold. ^a^ Units of Hounsfield Unit (HU) for CT value.

**Table 6:** Logistic regression predicting likelihood for differentiation PGIL from GIAC.

	B	S.E.	Wals	df	P	Odds Ratio	95%CI for Odds Ratio	
							Lower	Upper
**A-5%** **percentile**	−0.057	0.015	15.163	1	0.000	0.944	0.918	0.972
**V-median**	0.065	0.017	14.661	1	0.000	1.067	1.032	1.103
**Constant**	−3.124	1.021	9.360	1	0.002	0.044		

## Data Availability

The data presented in this study are available on request from the corresponding author. The data are not publicly available due to ethical.

## Supplementary Materials

The following are available online at https://www.mdpi.com/2075-1729/11/3/264/s1, Table S1: The interobserver agreement between two radiologists of different histogram parameters in both arterial and venous phase for patients with PGIL, Table S2: The interobserver agreement between two radiologists of different histogram parameters in both arterial and venous phase for patients with GIAC.
