# Supplementary material for: Primary Gastro-Intestinal Lymphoma and Gastro-Intestinal Adenocarcinoma: An Initial Study of CT Texture Analysis as Quantitative Biomarkers for Differentiation
Source: Life (Basel). 2021 Mar 23;11(3):264. doi: 10.3390/life11030264 (PMC8005065; doi:10.3390/life11030264)
Supplement: Supplementary file 1 [file life-11-00264-s001.pdf]

Table S1. The interobserver agreement between two radiologists of different histogram parameters in both arterial and venous phase for patients with PGIL.

Parameter	ICC	95%CI
A-mean	0.999	0.999 - 1.000
A-median	0.999	0.998 - 0.999
A-5%th percentile	0.998	0.997 - 0.999
A-10%th percentile	0.999	0.999 -1.000
A-25%th percentile	0.999	0.999 - 1.000
A-50%th percentile	0.999	0.999 - 1.000
A-75%th percentile	0.999	0.999 - 1.000
A-90%th percentile	0.999	0.998 - 1.000
A-95%th percentile	0.999	0.998 - 0.999
A-skewness	0.944	0.902 - 0.969
A-kurtosis	0.898	0.823 - 0.942
A-entropy	0.915	0.853 - 0.952
V-mean	0.998	0.997 - 0.999
V-median	0.998	0.997 - 0.999
V-5%th percentile	0.705	0.526 - 0.824
V-10%th percentile	0.715	0.540 - 0.830
V-25%th percentile	0.728	0.559 - 0.839
V-50%th percentile	0.756	0.601 - 0.857
V-75%th percentile	0.805	0.675 - 0.886
V-90%th percentile	0.852	0.749 - 0.915
V-95%th percentile	0.888	0.808 - 0.936
V-skewness	0.943	0.900 - 0.968
V-kurtosis	0.980	0.965 - 0.989
V-entropy	0.982	0.968 - 0.990

ICC, intraclass correlation coefficient; CI, confidence intervals

Table S2. The interobserver agreement between two radiologists of different histogram parameters in both arterial and venous phase for patients with GIAC.

**Parameter**	**ICC**	**95%CI**
A-mean	0.999	0.999 - 1.000
A-median	0.999	0.998 - 0.999
A-5%th percentile	0.998	0.997 - 0.999
A-10%th percentile	0.999	0.998 - 0.999
A-25%th percentile	0.998	0.998 - 0.999
A-50%th percentile	0.999	0.998 - 0.999
A-75%th percentile	0.999	0.998 - 0.999
A-90%th percentile	0.999	0.998 - 0.999
A-95%th percentile	0.999	0.998 - 0.999
A-skewness	0.909	0.857 - 0.942
A-kurtosis	0.826	0.735 - 0.888
A-entropy	0.890	0.829 - 0.930
V-mean	0.999	0.999 - 1.000
V-median	0.999	0.999 - 0.999
V-5%th percentile	0.998	0.996 - 0.999
V-10%th percentile	0.998	0.998 - 0.999
V-25%th percentile	0.999	0.998 - 0.999
V-50%th percentile	0.998	0.997 - 0.999
V-75%th percentile	0.997	0.996 - 0.998
V-90%th percentile	0.996	0.995 - 0.997
V-95%th percentile	0.997	0.995 - 0.998
V-skewness	0.978	0.965 - 0.986
V-kurtosis	0.961	0.938 - 0.975
V-entropy	0.856	0.779 - 0.908

ICC, intraclass correlation coefficient; CI, confidence intervals
